# Supplementary material for: Loss of PLA2G4E compromises synaptic structure and cognitive outcomes in mice
Source: Life Sci Alliance. 2025 Jul 9;8(9):e202503323. doi: 10.26508/lsa.202503323 (PMC12241665; doi:10.26508/lsa.202503323)
Supplement: Supplementary file 1 [file LSA-2025-03323_SdataF2_F5_F6_FS1_FS3_FS7_FS8.pptx]

## Slide 1
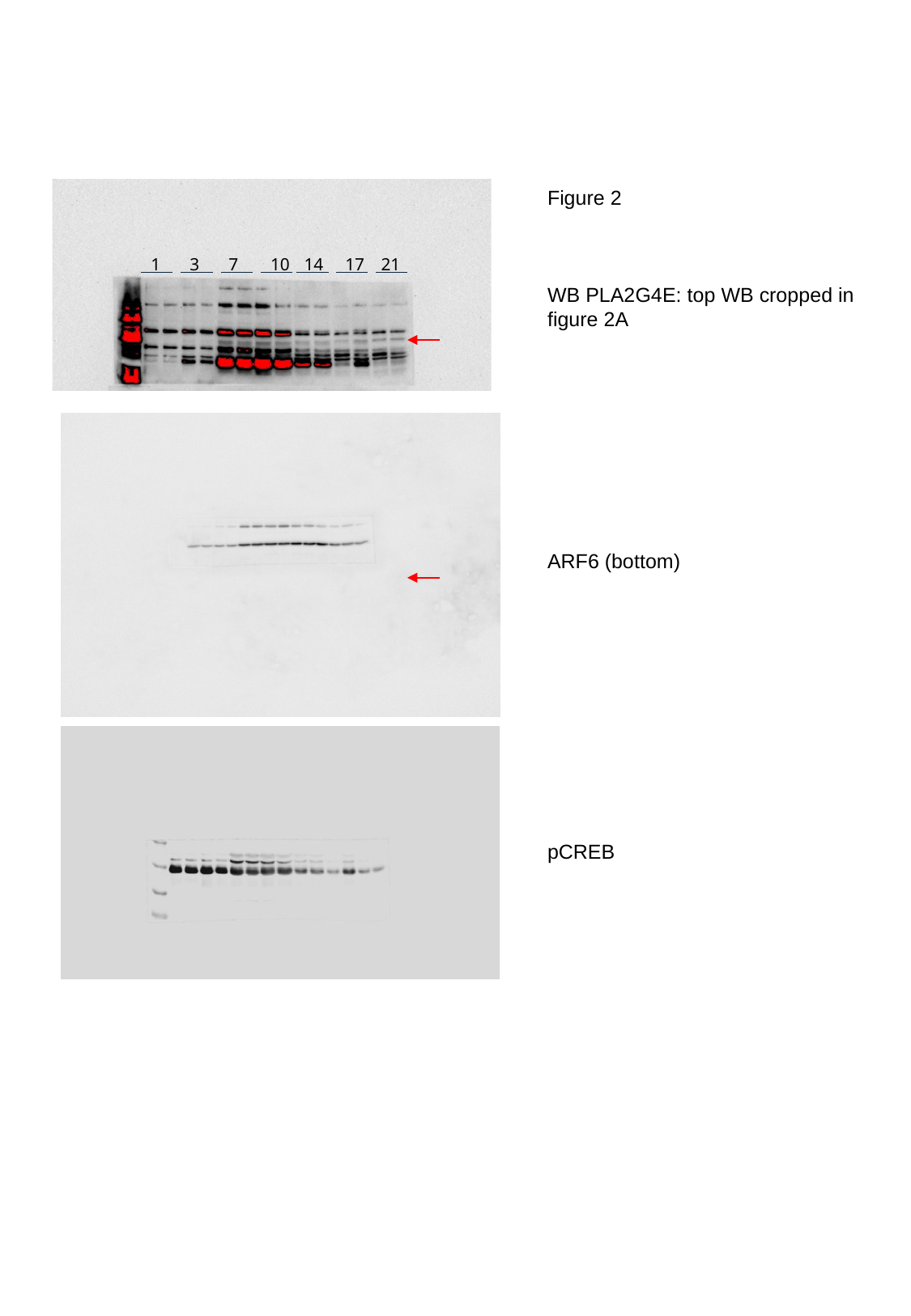

Figure 2
WB PLA2G4E: top WB cropped in figure 2A
ARF6 (bottom)
pCREB
1
3
7
10
14
17
21

## Slide 2
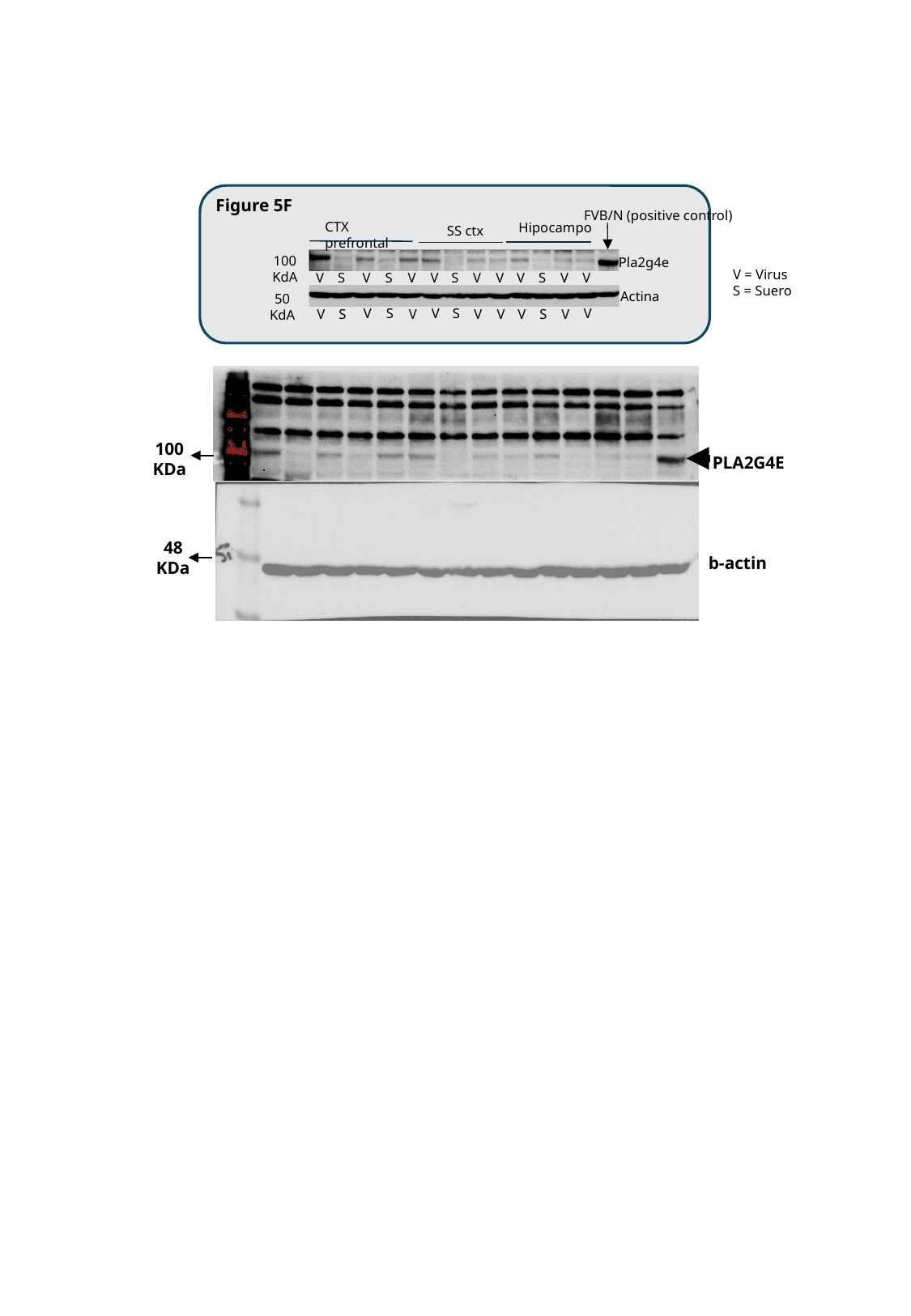

Figure 5F
FVB/N (positive control)
CTX prefrontal
Hipocampo
SS ctx
V
S
V
S
V
V
S
V
V
V
V
S
V
100 KdA
Pla2g4e
V = Virus
S = Suero
V
S
V
S
V
V
S
V
V
V
V
S
V
Actina
50 KdA
100 KDa
PLA2G4E
48 KDa
b-actin

## Slide 3
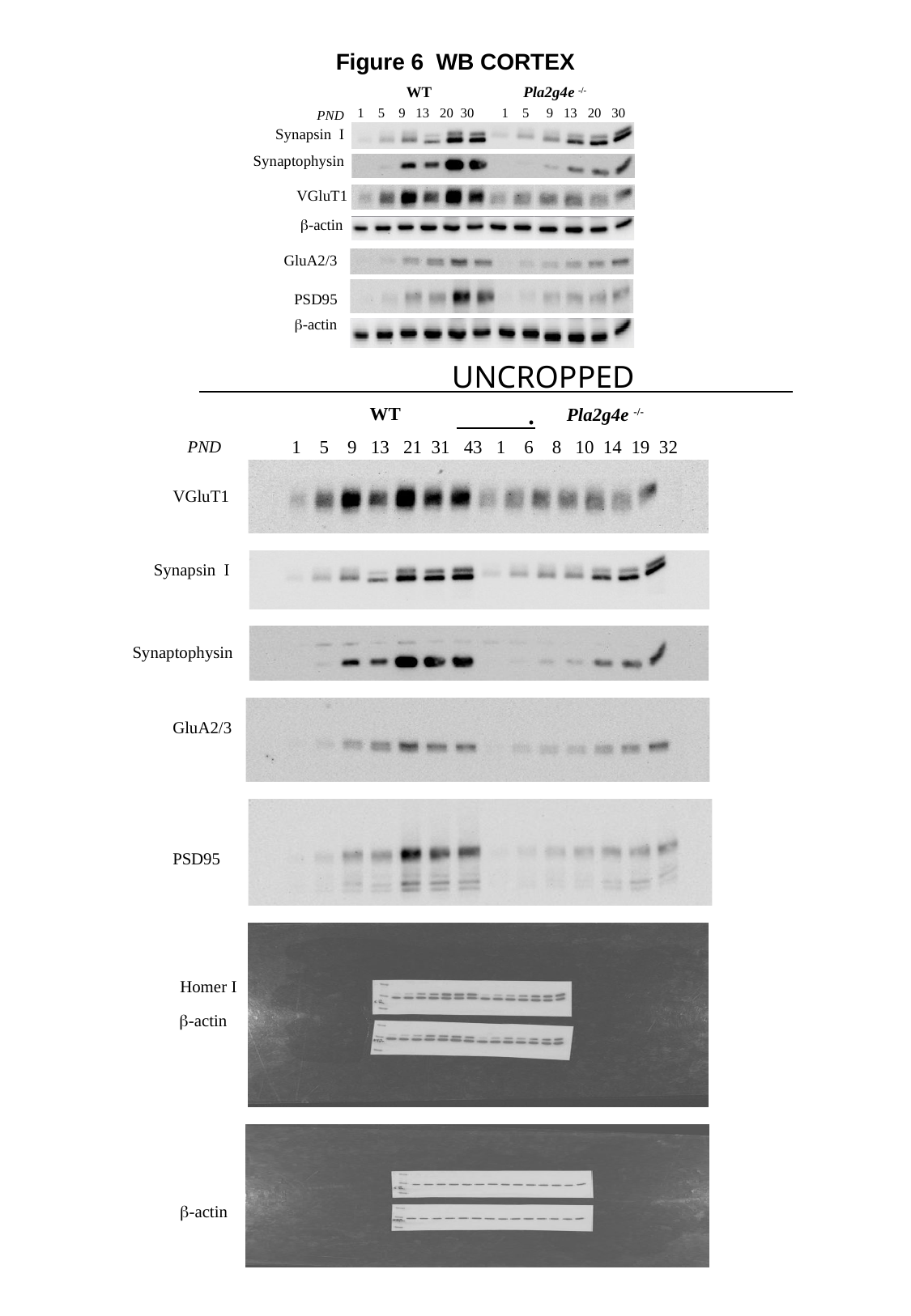

Figure 6 WB CORTEX
WT
Pla2g4e -/-
 1 5 9 13 20 30 1 5 9 13 20 30
PND
Synapsin I
Synaptophysin
VGluT1
b-actin
GluA2/3
PSD95
b-actin
 UNCROPPED .
WT
Pla2g4e -/-
 1 5 9 13 21 31 43 1 6 8 10 14 19 32
PND
VGluT1
Synapsin I
Synaptophysin
GluA2/3
PSD95
Homer I
b-actin
b-actin

## Slide 4
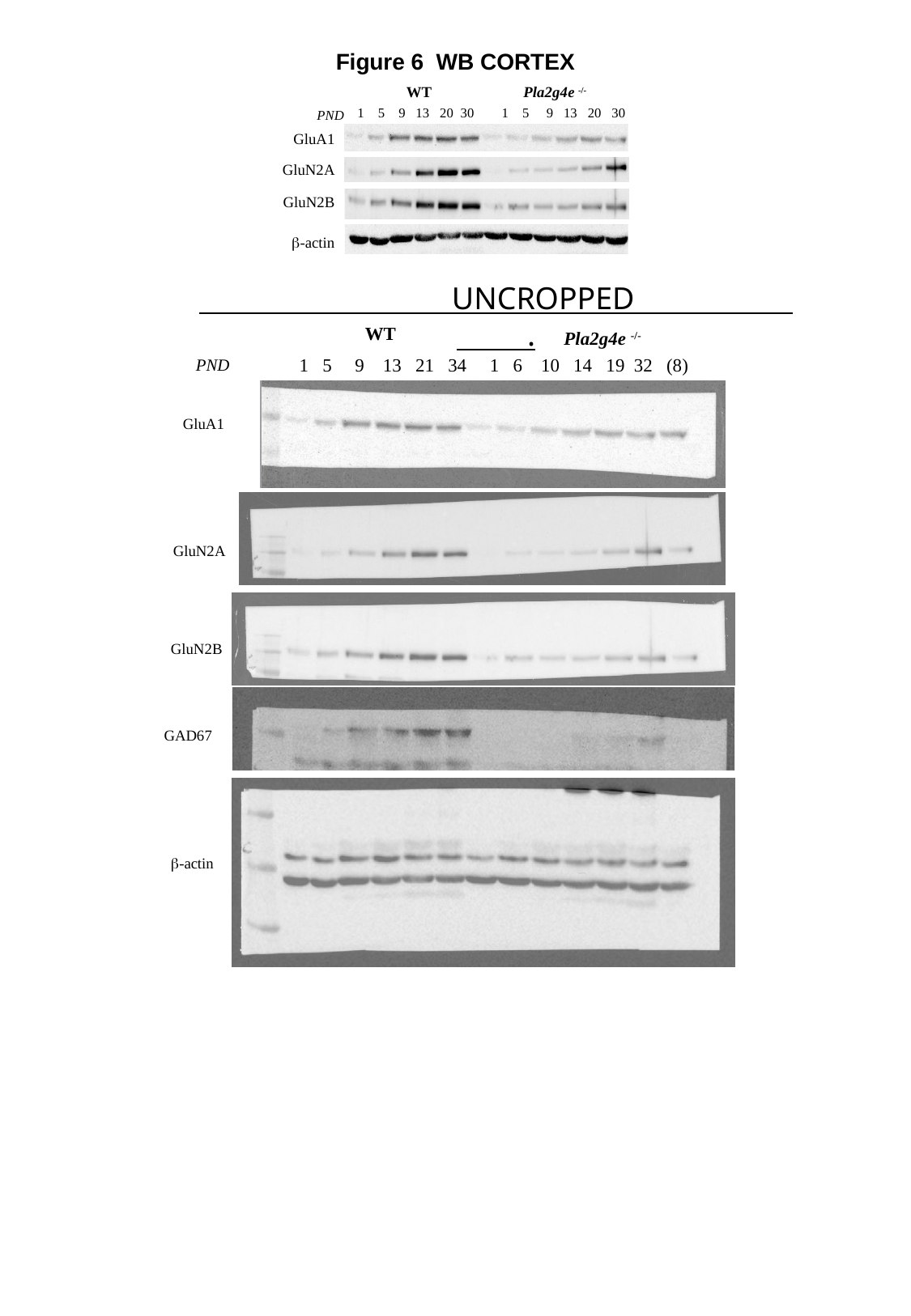

Figure 6 WB CORTEX
WT
Pla2g4e -/-
 1 5 9 13 20 30 1 5 9 13 20 30
PND
GluA1
GluN2A
GluN2B
b-actin
 UNCROPPED .
WT
Pla2g4e -/-
1 5 9 13 21 34 1 6 10 14 19 32 (8)
PND
GluA1
GluN2A
GluN2B
GAD67
b-actin

## Slide 5
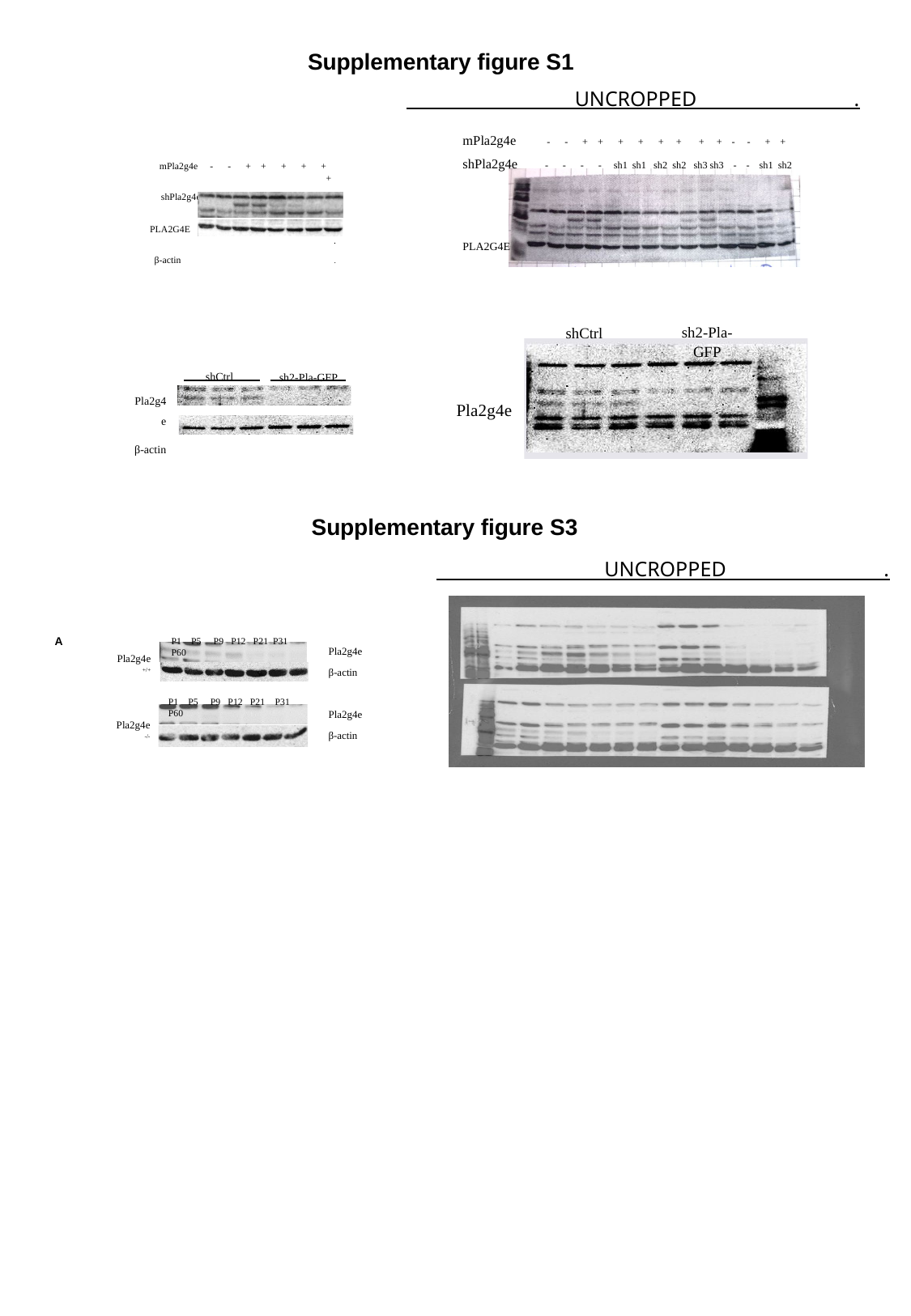

Supplementary figure S1
 UNCROPPED .
mPla2g4e - - + + + + + + + + - - + +
shPla2g4e - - - - sh1 sh1 sh2 sh2 sh3 sh3 - - sh1 sh2
PLA2G4E
mPla2g4e - - + + + + + + .
shPla2g4e - - - - sh1 sh1 sh2 sh2
PLA2G4E .
β-actin .
sh2-Pla-GFP
shCtrl
Pla2g4e
shCtrl
sh2-Pla-GFP
Pla2g4e
β-actin
Supplementary figure S3
 UNCROPPED .
A
P1 P5 P9 P12 P21 P31 P60
Pla2g4e
β-actin
Pla2g4e
β-actin
Pla2g4e +/+
P1 P5 P9 P12 P21 P31 P60
Pla2g4e -/-

## Slide 6
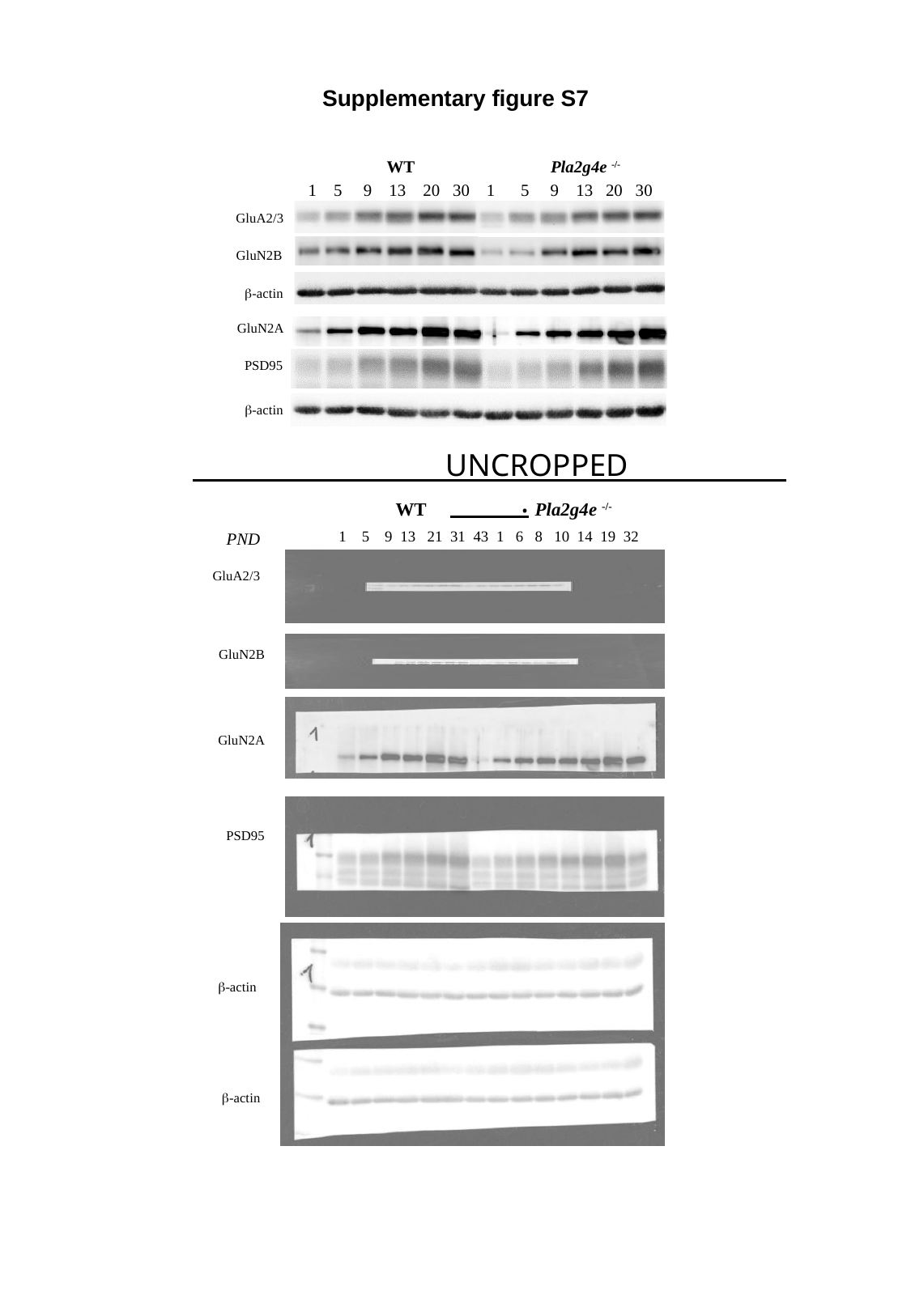

Supplementary figure S7
WT
Pla2g4e -/-
GluA2/3
GluN2B
b-actin
GluN2A
PSD95
b-actin
 1 5 9 13 20 30 1 5 9 13 20 30
 UNCROPPED .
WT
Pla2g4e -/-
 1 5 9 13 21 31 43 1 6 8 10 14 19 32
PND
GluA2/3
GluN2B
GluN2A
PSD95
b-actin
b-actin

## Slide 7
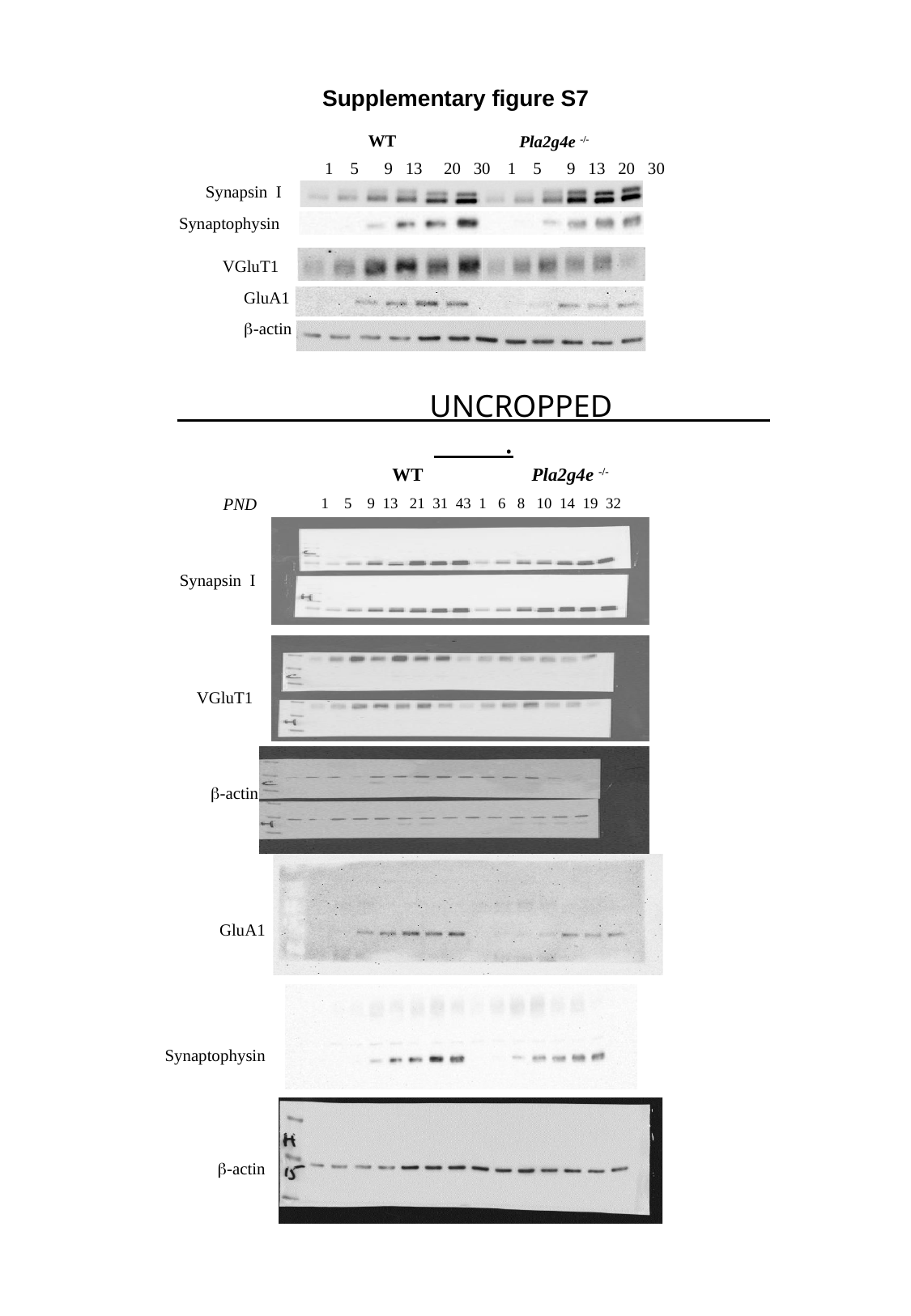

Supplementary figure S7
WT
Pla2g4e -/-
 1 5 9 13 20 30 1 5 9 13 20 30
Synapsin I
Synaptophysin
VGluT1
GluA1
b-actin
 UNCROPPED .
WT
Pla2g4e -/-
PND
 1 5 9 13 21 31 43 1 6 8 10 14 19 32
Synapsin I
VGluT1
b-actin
GluA1
Synaptophysin
b-actin

## Slide 8
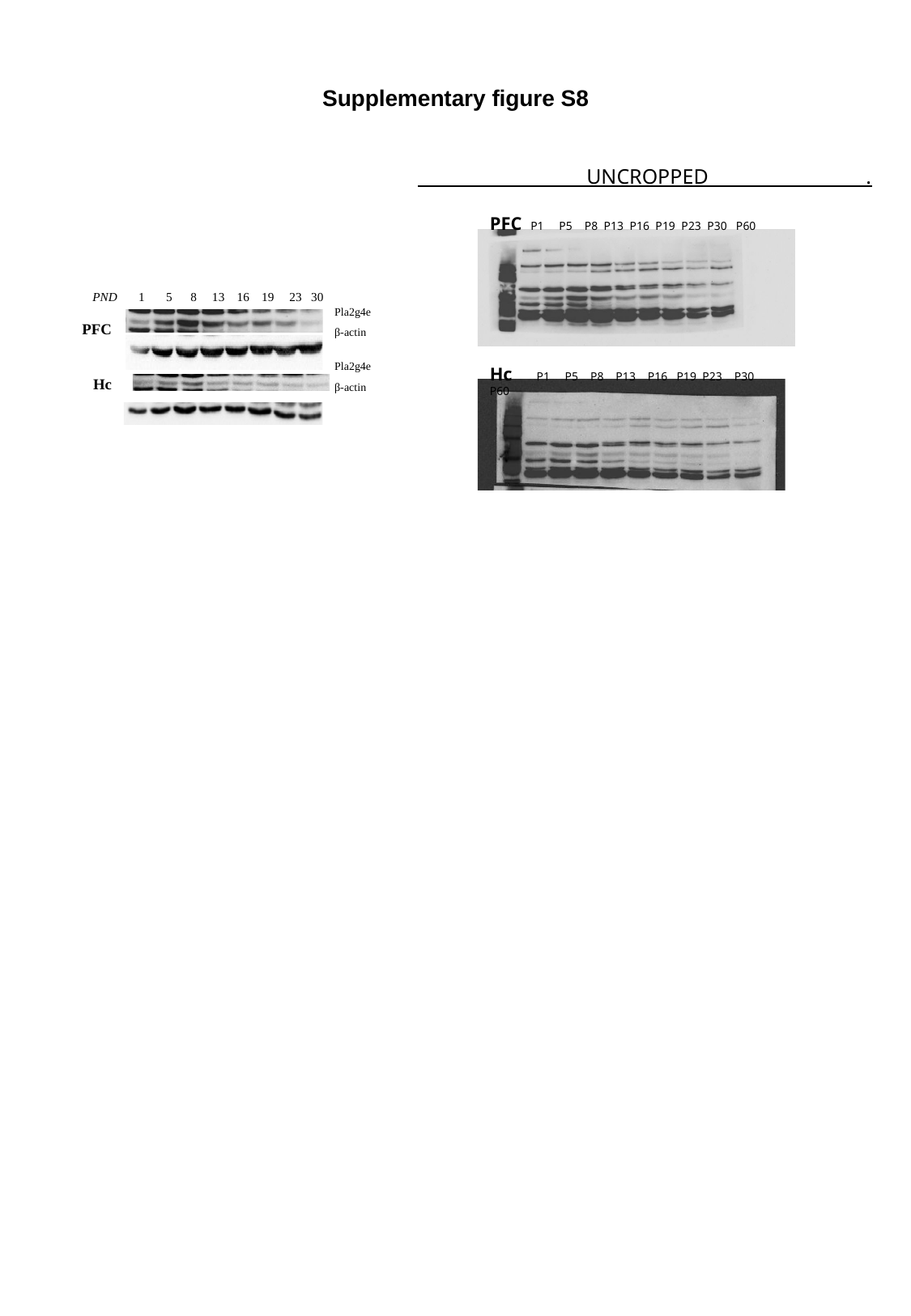

Supplementary figure S8
 UNCROPPED .
PFC P1 P5 P8 P13 P16 P19 P23 P30 P60
PND 1 5 8 13 16 19 23 30
Pla2g4e
β-actin
Pla2g4e
β-actin
PFC
Hc
Hc P1 P5 P8 P13 P16 P19 P23 P30 P60

## Slide 9
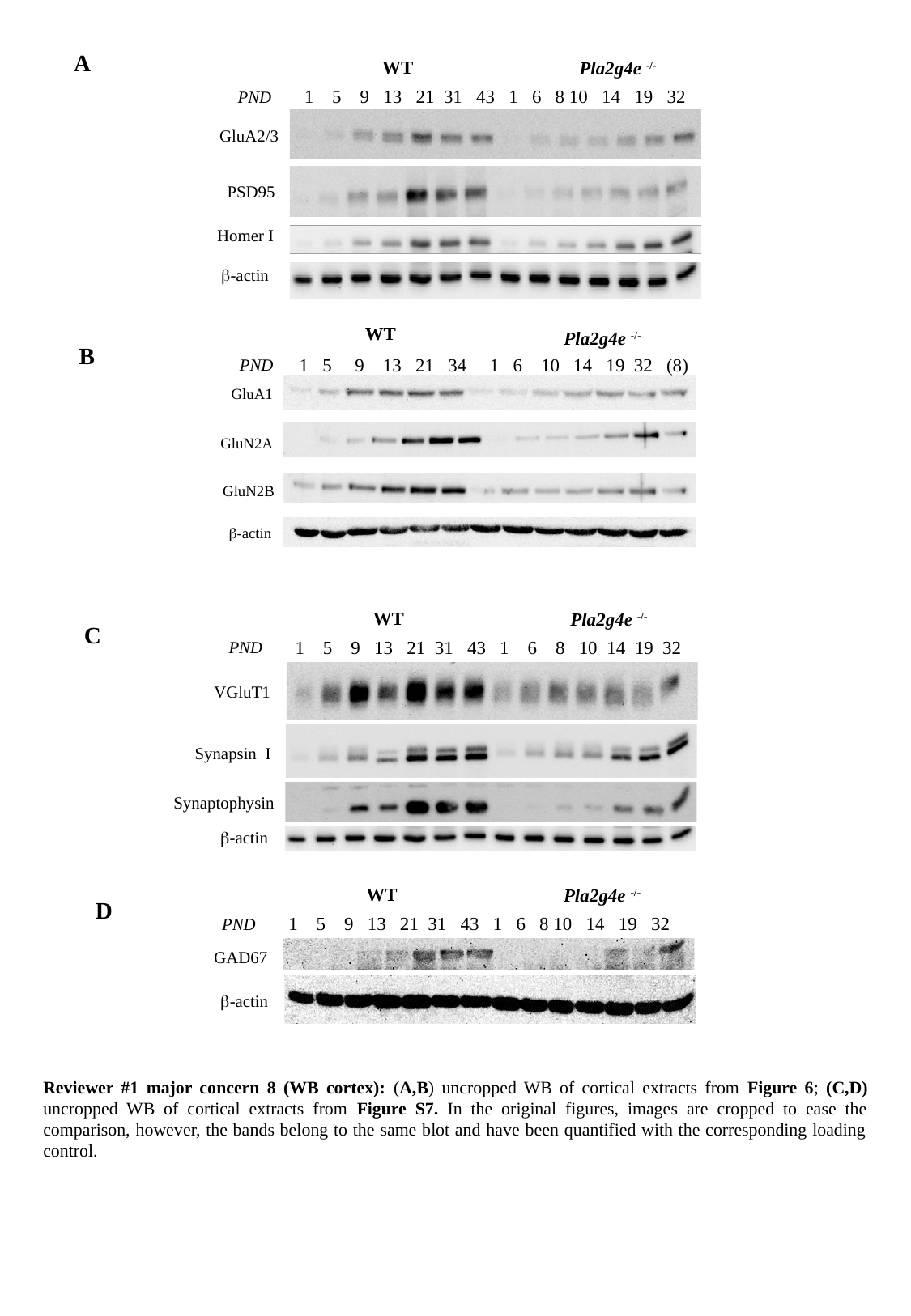

A
WT
Pla2g4e -/-
PND
GluA2/3
PSD95
Homer I
b-actin
 1 5 9 13 21 31 43 1 6 8 10 14 19 32
WT
Pla2g4e -/-
1 5 9 13 21 34 1 6 10 14 19 32 (8)
GluA1
GluN2A
GluN2B
b-actin
PND
B
WT
Pla2g4e -/-
C
 1 5 9 13 21 31 43 1 6 8 10 14 19 32
PND
VGluT1
Synapsin I
Synaptophysin
b-actin
WT
Pla2g4e -/-
D
 1 5 9 13 21 31 43 1 6 8 10 14 19 32
PND
GAD67
b-actin
Reviewer #1 major concern 8 (WB cortex): (A,B) uncropped WB of cortical extracts from Figure 6; (C,D) uncropped WB of cortical extracts from Figure S7. In the original figures, images are cropped to ease the comparison, however, the bands belong to the same blot and have been quantified with the corresponding loading control.
